# Importance of a Systematic Intervertebral Motion Parametrization for in vivo Assessment of Spine Biomechanics

**DOI:** 10.1007/s10439-025-03885-x

**Published:** 2025-11-20

**Authors:** Felix André Erb, Daniel Studer, Philippe Büchler, Carol-Claudius Hasler, Georg Rauter, Nicolas Gerig

**Affiliations:** 1 Department of Biomedical Engineering, University of Basel, Basel, Switzerland; 2 Orthopedic Department, University Children’s Hospital Basel (UKBB), University of Basel, Basel, Switzerland; 3 ARTOG Center for Biomedical Engineering Research, University of Bern, Bern, Switzerland

**Keywords:** Adolescent Idiopathic Scoliosis, in vivo Spinal Loading, Spinal Motion Parametrization, Functional Spinal Unit

## Abstract

**Purpose:**

Surgical decision-making for Adolescent Idiopathic Scoliosis (AIS) relies on geometrical rather than biomechanical properties, such as the spine’s in vivo load characteristics. While both in vivo and in vitro spinal loading experiments can provide valuable insights, a standardized method to compare motion for the Functional Spinal Unit (FSU) is lacking. This work aims to establish a systematic motion parametrization to unambiguously characterize FSU pose changes suitable for a robotic in vivo spinal loading application.

**Method:**

In this work, we propose an FSU motion parameterization using robotic rigid-body-tree modelling, deploying a virtual six-degree-of-freedom joint in the intervertebral space. To demonstrate the importance of the parameterization, we analysed the effect of different joint definitions on the produced displacement considering i) preoperative or intraoperative FSU reference poses, obtained from CT imaging of an AIS patient, and ii) one or both vertebral coordinate systems of the FSU. Additionally, we compared the required wrench capabilities of an actuation device to achieve pure spinal loading conditions in a pedicle screw-mounted scenario.

**Results:**

Applying identical virtual motions resulted in differences of up to 0.38 mm in translation and $24.19^{\circ }$ in rotation, depending on the joint definition. Corresponding required wrench capabilities showed maximum force and torque errors of up to 34.97$\%$ in virtual pure translation and bending experiments.

**Conclusion:**

Our findings underline the importance of a robust FSU kinematic framework, critical for ensuring safe and reliable FSU manipulation and for obtaining comparable and reproducible in vivo biomechanical data.

## Introduction

**Fig. 1:**
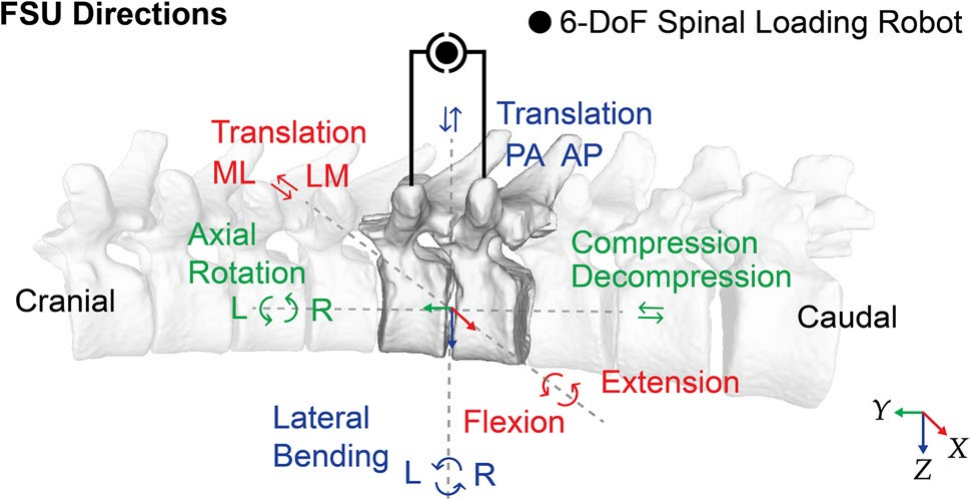
Spine segment (T5-L1) with the highlighted T10-T9 Functional Spinal Unit (FSU) and intervertebral coordinate system approximately aligned with physiological spinal directions, denoted by the following abbreviations: DoF, degrees of freedom; ML, medial–lateral; LM, lateral–medial; PA, posteroanterior; AP, anterioposterior; L, left; R, right. Schematic depiction of a 6-DoF spinal loading robot mounted parallel to the Spine Segment.

Corrective surgical decisions for deformities in Adolescent Idiopathic Scoliosis (AIS), such as determining which spinal levels require stiffening, have been based on simple biomechanical principles, empirical data from clinical studies, and, most importantly, surgeon experience. Quantifying surgeon experience has remained a challenge. Despite advances in the three-dimensional understanding of spinal deformities, Lenke’s classification, introduced in 2001  [1], has remained the standard for selecting fusion levels in corrective scoliosis surgery. This classification provides only limited, indirect insight into regional spinal flexibility, relying mainly on two-dimensional lateral bending radiographs. Knowledge of disease-specific ground-truth biomechanical properties for comparison is generally not available to clinicians during clinical assessments, such as probing the full range of motion and load-to-displacement behavior of spinal bending directions (e.g., Flexion/Extension, Axial Rotation Right/Left, and Lateral Bending L/R) (see Fig. 1).

Surgeries that are less invasive but sufficiently effective would require a detailed understanding of spine biomechanics. For example, the biomechanical properties of Functional Spinal Units (FSUs) need to be considered to decide which of the physiological motion units of the spine require stiffening through fusion or implant. To quantify FSU biomechanics, Spinal Loading Simulators (SLS) were used for in vitro evaluation of mono-segmental and poly-segmental load-to-displacement behavior in human and animal samples (e.g., [2–4]). Standardized SLS testing protocols for FSU were proposed, in which a cranial vertebra was deflected relative to a caudal vertebra [5, 6]. SLS setups comprised a load or displacement-inducing actuator capable of producing physiological loading scenarios in the FSU. Designs included orthogonal gantry systems [2, 3, 7–11], a serial robotic manipulator [12], or Stewart platforms [13, 14].

SLS and in silico studies reported different procedures to generate a joint coordinate system (JCS) around which an FSU motion is defined [15]: A JCS with origin in the facet plane of the cranial vertebra [7], the center of the cranial vertebra [14], or equidistant from the facet planes adjacent to the intervertebral space of the FSU [2, 16, 17] was used. Furthermore, some studies did not provide a clear definition of the JCS origin [9, 10, 12, 18, 19], for example, when imposing a force/torque profile and measuring the admitted displacement.

The choice of mappings, e.g., of 3D rotations with respect to 1D planar rotations, was shown to have a relevant impact on the resulting angles [20]. Nevertheless, we did not find studies on SLS that addressed the proper definition of motion directions of the JCS nor their impact on results relating to the JCS [3, 7, 9, 10, 17–19, 21, 22]. We therefore assumed that these studies based the JCS directions on the caudal vertebral coordinate systems as a recommended standard [15]. An alternative approach, utilizing the JCS directions derived from an interpolation of both the caudal and cranial vertebral coordinate systems, was proposed [16]. We identified the simplest approach to generating coordinate systems as being based on a landmark annotation. Studies have implemented proximal coordinates using four [15] or nine [23] landmarks for symmetrical vertebrae. Vertebrae of spines with deformities such as AIS typically showed skewness, distortions, and therefore asymmetry. Here, vertebra registration approaches with more landmarks  [24] or surface detection [25] would allow for capturing asymmetric vertebrae shapes more effectively.

The standard practice in SLS experiments involves pure bending conditions, which imply the exclusive application of a torque around one principal axis of the JCS. Therefore, such pure bending SLS experiments by design rely on the JCS origin and direction definitions. Alignment was typically performed manually [2, 21]. However, these SLS studies did not explore the definition of JCS directions and their implications for the displacement or load experiments performed. Inconsistent alignment would introduce apparatus-induced artifacts. To alleviate alignment challenges, free translation of the caudal vertebra was realized in bending experiments by minimizing friction, which allowed the JCS to self-adjust and increased reproducibility in SLS studies  [19]. However, we considered that such a free-floating caudal vertebra solution might not reflect physiological FSU properties sufficiently. Besides the spinal disk and ligaments that are present in SLS experiments, the surrounding tissues of an FSU (e.g, muscles, connective tissue) could affect FSU biomechanics (e.g, stiffness). Furthermore, a definition of JCS directions based on the coordinate system of one vertebra could be limiting in case of a strongly nonsymmetrical or skewed vertebra. Therefore, we are convinced that a common and unambiguous definition for vertebra coordinate systems and JCS that completely parameterizes the FSU motion would be needed.

We identified two major measurement regimes for load/displacement experiments with causally controlled devices such as SLSs or spinal loading robots. i) In a mechanical admittance regime, the device enforces a defined wrench profile $\vec {w}(t)$ (i.e., force and torque) while recording the admitted motion [2, 3, 6, 9, 10, 18, 19, 22]. ii) In a mechanical impedance regime, the device enforces a defined motion profile (e.g., represented as the transform from the caudal $V_1$ to the cranial $V_2$ vertebra coordinate system) while recording the impeding wrench [4, 8, 10–12, 14, 21]. Throughout this paper, the terms impedance and admittance exclusively refer to mechanical impedance and mechanical admittance.

Further measurement regimes would be possible while recording both the resulting wrench and motion, e.g., random perturbations or any form of compromise between following a desired wrench and motion profile simultaneously. Manual application is an example of such a measurement regime with an unknown compromise between following a desired wrench and motion profile arising from the individual human operators and their motor control  [22, 26–29]. We limited ourselves to pure impedance and admittance regimes, that we considered more reproducible. During an impedance or admittance measurement, a discrepancy between the expected and perceived motion or wrench can be detected and corrected, further making such measurements safer for the patient.

Progress in the field of in vitro spine biomechanics for diseases such as AIS was slow due to the scarcity of body donors with untreated, non-degenerative pathologies. In vivo FSU measurements during corrective surgery could provide a unique opportunity to assess the load-to-displacement behavior in pathological FSUs directly. Reported instrumented force devices, such as force-sensing distraction forceps, measured a force–displacement relationship with limited force orientations on degenerative FSUs  [22, 26–28] and AIS FSUs [29]. Experiments are based on SLS principles; the need for coordinates and excitation directions remains open, which supports the need for an anatomy-specific standard. Pure bending moments are only applied to the JCS if alignment with the SLS is achieved. However, manually mapping the tool frame to the JCS frame is error-prone and may result in stray forces in undesired directions. Because 3D rotations are not commutative, rotations deviating from a single axis cannot be retraced. Furthermore, patient repositioning may lead to an equilibrium state of the FSU that is different before and during measurement. Also, effects of muscle relaxation or surgical dissection (e.g., ligaments, muscles, or joints) were typically not considered  [22, 26–28]. Previous work in tracking changes in the pose of the scoliotic FSU from preoperative to intraoperative state revealed a relevant displacement of the vertebrae’s relative position and orientation [25]. This affects the quality of comparisons or combinations across in vivo experiments and in vitro SLS studies.

Instead of manual in vivo FSU assessments, robotic approaches could overcome reproducibility challenges while leveraging the benefits of closed-loop control that SLS measurements also offer. Robotic in vivo testing would require the following: 1) a strictly defined JCS based on the FSU’s anatomy and resting position and 2) a device that can precisely control the execution of a desired motion or wrench profile and measure wrench and pose to realize measurements in either an impedance or admittance control regime. To our knowledge, the only robotic spinal loading system that targeted deployment in vivo for such measurements was the SpineBot [18]. However, this device did not reach technological readiness for in vivo FSU assessments. A procedural FSU convention is beneficial because of the need for a unambiguous definition of a kinematic motion parameterization that could facilitate controlled manipulation of an FSU by a robotic device. To our understanding, a generalizable method for FSUs of typical and atypical shapes would enhance the ability to compare the results of SLS studies, previous handheld in vivo studies, and future studies, including those involving robotic devices.

The aims of the present study were threefold:

1. To develop a convention to quantify changes in the pose of the FSU in 6-DoF suitable for robotic applications: For this, we assumed that vertebral bodies can be treated as rigid bodies, i.e., they do not deform significantly when the FSU pose changes. Furthermore, we assumed that a meaningful JCS for pure bending is defined at the center of the intervertebral space of the FSU.
2. To demonstrate the implications of this convention by analyzing virtual single-axis displacements of the FSUs and testing two hypotheses:

### Hypothesis 1

Defining the JCS based on either the preoperative or intraoperative FSU pose results in relevant differences in the produced virtual motion, assessed on the location of the deflected vertebra. For this, we evaluated the vertebrae shift of two FSUs from an AIS patient in the preoperative and intraoperative states before and during corrective surgery.

### Hypothesis 2

Defining the JCS using either the caudal vertebra alone or both the caudal and cranial vertebrae produces relevant differences in virtual motion.

3) To analyze the required wrench capability (force/torque) of a hypothetical in vivo spinal measurement device to perform the derived virtual motions above by testing a third hypothesis:

### Hypothesis 3

The required wrench capabilities differ depending on whether the JCS is defined based on the preoperative or intraoperative FSU pose, and whether it is referenced to the caudal vertebra alone or to both caudal and cranial vertebrae.

Here, we assumed static mounting of pedicle screws for the application points of the wrench, similar to SpineBot [18], and relied on the pedicle screw entry position from a preoperative planning of the AIS patient.

**Hypothesis 2** and **Hypothesis 3** were evaluated under consideration of a vertebral shift from the preoperative to the intraoperative state.

## Material and Methods

### Patient Data

Preoperative (T5-L1) and intraoperative (T12-T8) CT Scans from one patient (f, 16y), showing a characteristic severe progression of AIS, were obtained from the Orthopedic and Radiology Department at the University Children’s Hospital Basel (UKBB), University of Basel, Switzerland. The surgical access to the spine was performed using a standard posterior midline approach with subperiosteal preparation of the posterior elements of the spinal column. After dissection, an intraoperative CT scan was conducted to navigate pedicle screw insertion. T8 and T12 were partially resolved. Polyaxial VERSE DePuy Synthes Pedicle screws with extended tabs (DePuy Synthes, Raynham, MA, USA) were used in various diameters and lengths during surgery. Both the preoperative and intraoperative scans were segmented in parts using the segmentation software Customy Vision (Smart Labs Sp. z o.o., Chorzów, Poland) and in-house segmentation software.

### FSU Motion Parametrization

**Fig. 2:**
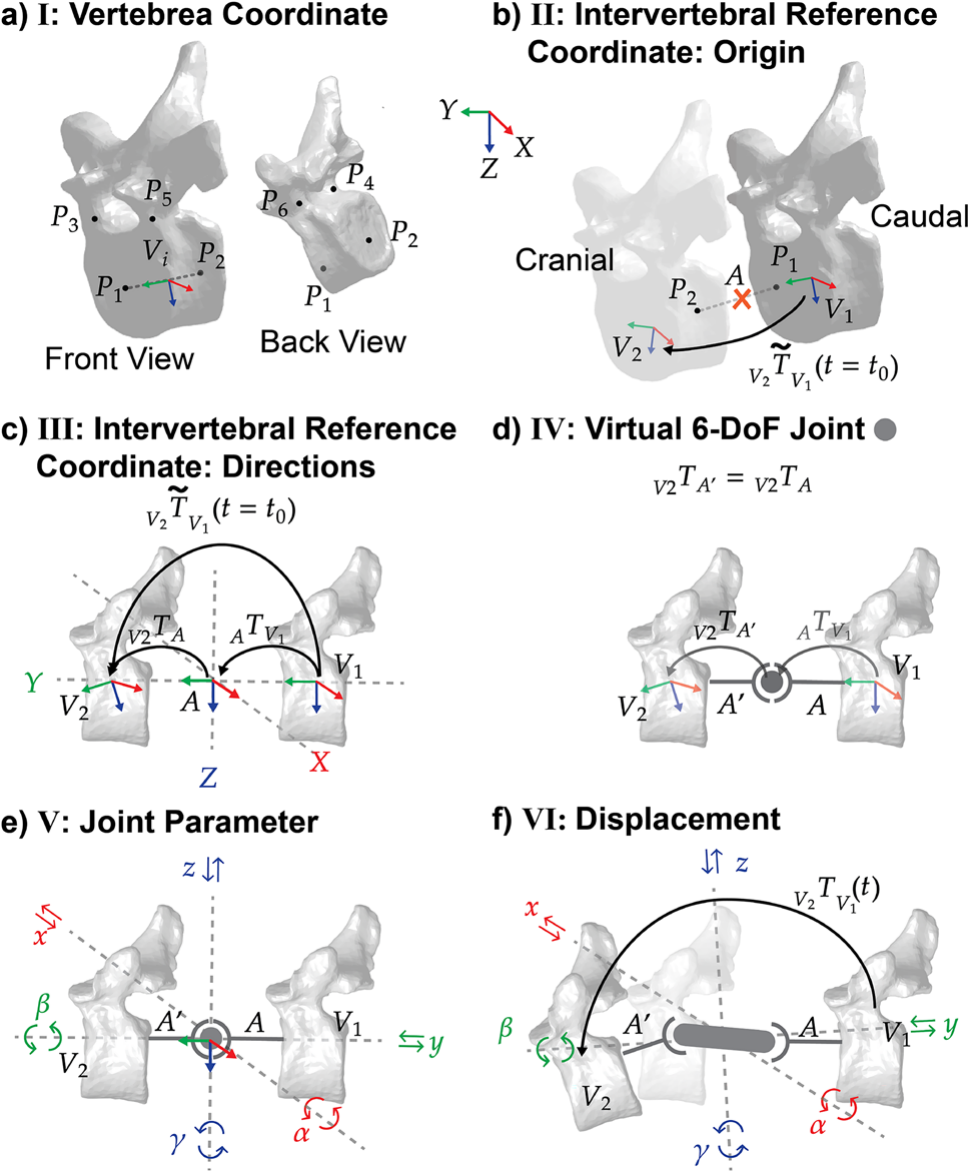
Systematic Intervertebral Motion Parametrization: **a**) Step *I*: Vertebra coordinate system definition based on 6 landmark registration ($P_{1...6}$) to define the origin of a vertebra coordinate system $V_i$ (Midpoint of dashed line of $\overline{P_1P_2}$). **b)** Step *II*: Placement of Intervertebral reference coordinate system *A* (orange cross) at the midpoint between $P_1$ of caudal vertebra  $V_1$and $P_2$ of cranial vertebra $V_2$). The transform $_{V_2}\tilde{T}_{V_1}(t)$ is defined accordingly. **c)** Step *III*: Orientation of the Intervertebral reference coordinate system *A*. Depicted case: caudal vertebra coordinate system orientation as reference for the JCS. The reference time point transform $_{V_2}\tilde{T}_{V_1}(t_0)=const.$ (measured from independent vertebra registration) is split into two static transforms $_{V_2}T_{A}=const.$ and $_{A}T_{V_1}=const.$ **d)** Step *IV*: Virtual 6-DoF robotic joint $\bullet$(t) between *A* and $A'$ (rigid body after joint) is introduced. $_{V_2}T_{A'}$ = $_{V_2}T_{A}$. **e)** Step *V*: Joint parameters $x,y,z,\alpha ,\beta , \gamma$ are introduced, which reflect the physiological motion directions stated in Fig. 1. **f)** Step *VI*: FSU pose change is characterized by joint parameters.

Vertebrae were registered using six landmarks, adapted from the nine-point scheme of André et al. [23], which was originally defined for radiographic visibility. Since CT-segmented data are not subject to visibility limitations, we excluded posterior landmarks and focused on the vertebral body and pedicles. This provided stable, body-centered registration points while avoiding features prone to deformities (e.g., transverse and spinous processes) or alterations during surgery (e.g., facet joints). The selected landmarks were defined as follows (Fig. 2a): ($P_1$) center of the cranial endplate, ($P_2$) center of the caudal endplate, ($P_3$) high point of cranial pedicle right, ($P_4$) low point of cranial pedicle right, ($P_5$) high point of cranial pedicle left, and ($P_6$) low point of cranial pedicle left.

Annotation was performed on the corresponding preoperative mesh surface using the 3D Slicer’s Landmark Registration Tool (Surgical Planning Laboratory, Boston, USA).

#### Vertebra Coordinate System

The vertebral center location (origin) $\vec {r}_{O_{V_{i}}}$ of vertebra $V_{i}$ was:

$$\begin{aligned} \vec {r}_{O_{V_{i}}} =\vec {r}_{P_{1}} + \frac{1}{2}\overline{P_1 P_2} \end{aligned}$$

To account for skewness and asymmetry of a scoliotic vertebra, we defined the root coordinates for $V_1$ and $V_2$ as $f_{Coord}(\vec {r}_{O_{V_1}},\vec {a}_{V_1},\vec {b}_{V_1})$ and $f_{Coord}(\vec {r}_{O_{V_2}}\vec {a}_{V2}, \vec {b}_{V2},)$ with $\vec {a}_{Vi} = \overline{P_2P_1}$ and $\vec {b}_{Vi} = \frac{1}{2}(\overline{P_3P_5}+\overline{P_4P_6})$ of the respective vertebra.

As an example: We constructed the orthogonal coordinate system of $V_1$ relative to the origin coordinate frame *O* with the three input vectors ($_{O}\vec {r}_{O_{V_1}}$,$_{O}\vec {a}_{V_1}$,$_{O}\vec {b}_{V_1}$) using the following procedure:

$f_{Coord}({_{O}\vec {r}_{O_{V_1}}},{_{O}\vec {a}_{V_1}},{_O\vec {b}_{V_1}}) = ({_O\vec {e}_{x_{V_1}}},{_O\vec {e}_{y_{{V_1}}}},{_O\vec {e}_{z_{{V_1}}}})$, where unit vector axis directions are defined as follows:

$$\begin{aligned} {_O\vec {e}_{Y_{V_1}}}&= \dfrac{_O\vec {a}}{\Vert _O\vec {a}\Vert }, \quad {_O\vec {e}_{Z_{V_1}}} = \frac{_O\vec {b}}{\Vert _O\vec {b}\Vert } \times {_O\vec {e}_{Y_{V_1}}}\\ {_O\vec {e}_{X_{V_1}}}&= {_O\vec {e}_{Y_{V_1}}} \times {_O\vec {e}_{Z_{V_1}}} \end{aligned}$$

With this procedure, we aligned the local vertebra coordinate of $V_i$ as described by Panjabi et al. (1976) [5], such that unit vectors X, Y, and Z are left, cephalad, and anterior, respectively.

#### Intervertebral Reference Coordinate System

We will refer to the coordinate system of the FSU’s joint as the “Intervertebral Reference Coordinate System.” This coordinate system marks the reference for a virtual joint in the kinematic chain. We defined the Intervertebral Reference Coordinate System origin ${O_A}$ relative to the coordinates ${V_{1}}$ and/or ${V_{2}}$, choosing equidistant origin placement from $P_2$ of vertebra $V_1$ and $P_1$ of vertebra $V_{2}$ (Fig. 2b). Figure 2b displays a case in which the caudal vertebra is taken as reference and the axis directions of *A* are kept equal to those of $V_1$.

A rigid body tree model was constructed using MATLAB’s Robotics Toolbox (MathWorks, Natick, MA, USA) (Fig. 2c). We denote $t_0$ as the time of taking the medical image for the initial position. Using the coordinate systems ${\displaystyle V_{1}}$ and ${\displaystyle V_{2}}$ , we can obtain the homogeneous transformation ${\displaystyle _{V2}\tilde{T}_{V_{1}{}}(t_0)}=const.$ that is split in the transform $_{A}T_{V_1}=const.$ and $_{V_2}T_{A}=const.$

We use the following notation: The location expressed in coordinate frame $V_1$ (${_{V_1}\vec {r}} = (_{V_1}r_x, _{V_1}r_y, _{V_1}r_z, 1)^T$) can be expressed in coordinate frame $V_2$ by left multiplication ${\vec {_{V_2} r} = {_{V_2}T_{V_1}} \cdot {_{V_1}\vec {r}}}$ of the transformation matrix from $V_1$ to $V_2$ is of the form:

$$\begin{aligned} {\displaystyle _{V_2} T_{V_1} \ =\ \begin{pmatrix} \begin{matrix} {_{V_2}R_{V_1}} \end{matrix} \ & {_{V_2}\vec {r}_{O_{V_1}}}\\ \begin{matrix} 0 & 0 & 0 \end{matrix}&\begin{matrix} 1 \end{matrix} \end{pmatrix}} \end{aligned}$$

where ${_{V_2}R_{V_1}}$ is the rotation from $V_1$ to $V_2$ expressed in $V_2$, and ${_{V_2}\vec {r}_{O_{V_1}}}$ is the translation of $V_1$ with respect to the origin to $V_2$ expressed in $V_1$.

#### Virtual 6-DoF Joint

We introduced a virtual 6-DoF Joint $$\displaystyle \bullet \ $$ (Fig. 2d) that allows fully parametrized motion between the vertebrae at the location of the Intervertebral Reference Coordinate System between ${\displaystyle A}$ and defined a reference system ${\displaystyle A'}$ directly after the virtual joint. The coordinate system *A* before the joint moves with vertebra $V_1$ ($_{A}T_{V_1}$). The coordinate system *A* after the joint moves with vertebra $V_2$ ($_{V_2}T_{A}=_{V_2}T_{A}$). All motions between the two vertebrae are expressed with the virtual joint $$\displaystyle \bullet \ $$. We defined the joint coordinates ${\vec {\theta }(t)}$ and assigned them to the physiological motion directions of the FSU, as illustrated in Fig. 2d). These six joint parameters are analogous to the physiological motion parameters. We considered such a motion parameterization to be the most intuitive for communication with our medical experts. The rigid body joint $A\Rightarrow A'$ was constructed as a serial joint of three prismatic X-Y-Z joints and three revolute joints $\alpha _X, \beta _Y, \gamma _Z$, as shown in Fig. 2c. Throughout this work, we denote the combination of the intervertebral reference system and 6-DOF joint as JCS. The 6 parameters Fig. 2e) defining the FSU motion written in vector notation are then:

$${\vec {\theta }(t) = (\theta _{x}(t),\theta _{y}(t), \theta _{z}(t),\theta _{\alpha }(t),\theta _{\beta }(t),\theta _{\gamma }(t))^T}$$.

The transform ${\displaystyle _{A'} T_{A} (\vec {\theta } (t))}$ for the 6-DoF joint results as:

1 $$\begin{aligned} \displaystyle _{A'} T_{A}(\vec {\theta })= {\displaystyle \begin{pmatrix} R_{z} (\theta _{\gamma }) R_{y} (\theta _{\beta }) R_{x} (\theta _{\alpha }) & \theta _{x}\\ & \theta _{y}\\ & \theta _{z}\\ \ 0\ \ 0\ \ 0 & 1 \end{pmatrix}} \end{aligned}$$

where at $$t = t_0$$:

$${\displaystyle \overrightarrow{\theta } (t_0) \equiv 0 \Rightarrow {_{A'}T_{A}} (0) = \begin{pmatrix} I_{3\times 3} & \vec {0} \\ \vec {0}^{T} & 1 \end{pmatrix}}$$

With this convention, the FSU is fully parameterized at the origin of the JCS. Any introduced displacement through vertebral shift or spinal loading is parameterized with the virtual joint parameters $\vec {\theta }(t>t_0)$ (Fig. 2f).

In this work, we considered and evaluated two alternative definitions of JCS directions to assess their implications on produced FSU motion and the required wrench capability:

- *Single Parent*: For simplicity and intuitiveness, the caudal vertebra is taken as reference and axis directions of *A* are kept equal to those $V_1$: ${_{V_1}R_A}:= I_{3\times 3}$, ${{_IR_{A}} = {_IR_{V_1}}}$. Where *R* and *I* denote the rotational and identity matrices, respectively. This seemed to be the standard in SLS experiments.
- *Double Parent*: For a definition that is less dependent on the configuration at reference construction, the axis directions of $A$ were computed by interpolating between those of $V_1$ and $V_2$ using spherical linear interpolation (SLERP). Specifically, the rotation from $V_1$ to $V_2$ was first expressed as a quaternion $\textbf{q}$. Then an interpolated quaternion was computed as:
  $$\vec {q}_A:= \text {slerp}(\vec {1}, \vec {q}, r),$$
  where $\vec {1}$ is the identity quaternion and $r \in [0,1]$ the interpolation ratio (e.g., $r = 0.5$ for equal contribution). The corresponding rotation matrix $R_A$ was then obtained via conversion from $\vec {q}_A$. As before, we define
  $$_{V_1}R_A = _A R_{V_2}:= R_{\text {const}}, \quad \text {with} \quad R_{\text {const}}^2 = _{V_1}R_{V_2}.$$
  We assumed the *Double Parent* definition to be a candidate for FSUs with strongly skewed and asymmetric vertebrae as found in AIS.

### FSU Reference: Preoperative to Intraoperative

**Fig. 3:**
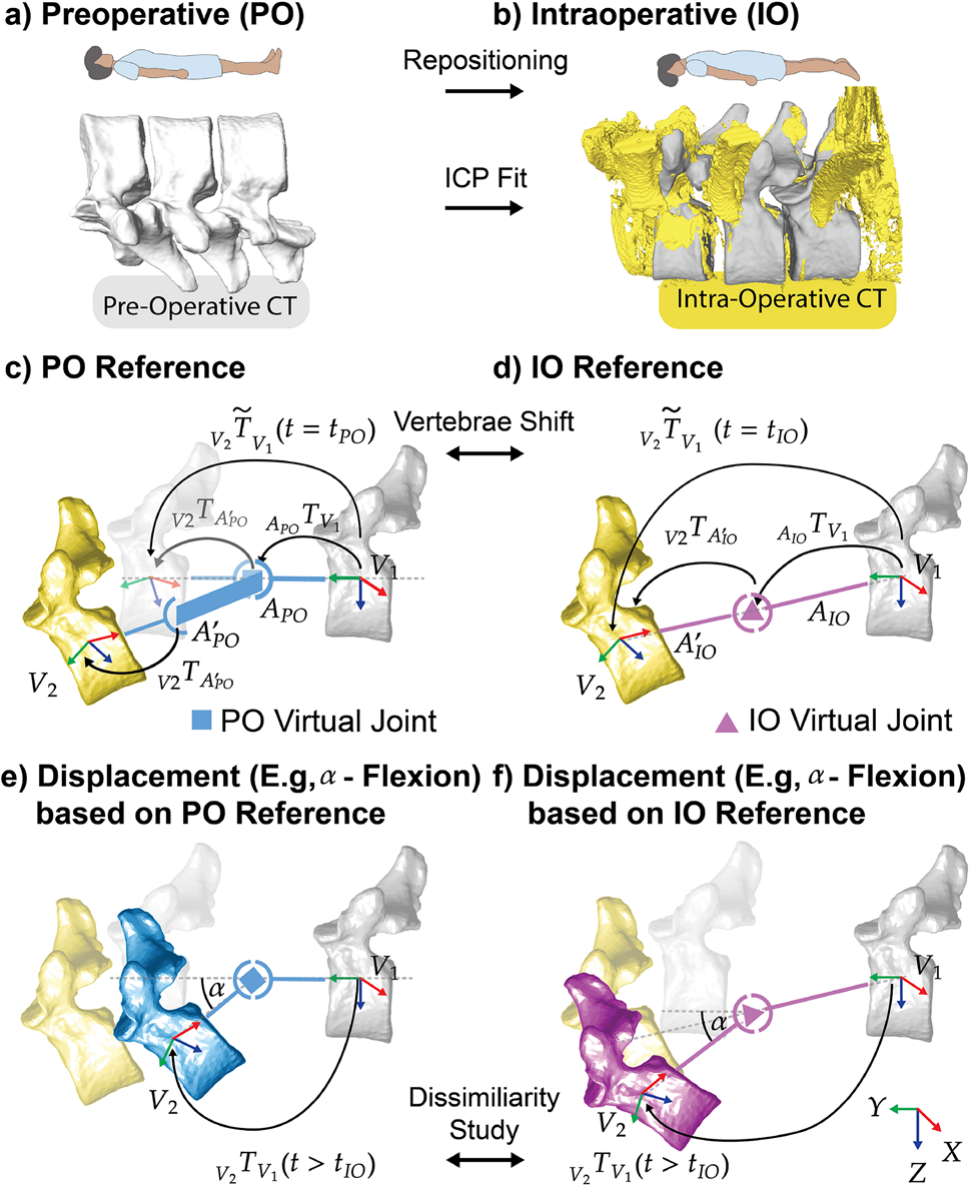
FSU reference choice and its effect on spinal displacement: **a**) The FSU pose in the preoperative configuration is recorded using CT in the supine position. **b**) FSU pose in the intraoperative configuration is recorded using C-arm CT in the prone position. Preoperative high-resolution segmented meshes (gray) are fitted to segmented meshes of intraoperative scans (yellow) using an ICP mesh fitting. The study comprises FSUs T9–T10 and T10–T11 from one AIS patient. **c**) Following the systematic convention, we generate a 6-DoF virtual joint $\blacksquare$ (blue) based on measured $_{V_{2}}\tilde{T}_{V_{1}}(t_{PO})$, taking the preoperative configuration as reference pose. An intraoperative FSU configuration is expressed with PO virtual joint as explained in Sect. 2.2.3. **d**) Similarly, we generate a 6-DoF virtual joint $\blacktriangle$ (magenta) based on $_{V_{2}}\tilde{T}_{V_{1}}(t_{IO})$, taking the intraoperative configuration as reference pose. Vertebra shift from PO to IO based on $_{V_{2}}\tilde{T}_{V_{1}}$ is conducted in Sect. 2.4. **e**) Depicted displacement (e.g., $\alpha$-Flexion) of the intraoperative configuration, showing the displacement of vertebra $V_2$ (yellow) based on the PO reference pose, resulting in the blue vertebra position. **f** Depicted displacement (e.g., $\alpha$-Flexion) of the intraoperative configuration, showing the displacement of vertebra $V_2$ (yellow) based on the IO reference pose, resulting in the magenta vertebra position. PO (blue) and IO (purple)–based displacement positions of $V_2$ are subjected to a dissimilarity analysis based on $_{V_{2}}\tilde{T}_{V_{1}}(t > t_{IO})$. (Anatomical positions: Copyright Evelyn Bailey, downloaded from thoughtco.com.).

A reference FSU pose is required to define the Intervertebral Reference Coordinate system and derive FSU pose changes. Typically, the preoperative configuration is captured via CT in supine position for surgical planning (Fig. 3a). The intraoperative configuration, imaged via C-arm CT in prone position, differs due to patient positioning and anatomical alterations, such as surgical dissection (Fig. 3b). In the case of FSU poses, such an intraoperative pose change from preoperative to intraoperative configuration may be considerable. A careful choice of FSU reference is required because the reference pose determines the zero-displacement configuration when assessing mechanical force–displacement relationships.

The intraoperative configuration at time $t_{IO}$ represents the spinal pose prior to external load application. The transformation ${}_{V_2}\tilde{T}_{V_1}(t_{IO})$ quantifies the FSU pose, which can be expressed using the preoperatively defined joint coordinate system $A_{PO}$ with parameters $\vec {\theta }_{PO}(t_{IO}) \not \equiv 0$ (Fig. 3c). This configuration defines the equilibrium pose used for stiffness assessment. The subindex *PO* indicates that the virtual 6-DoF joint placement (denoted as $\blacksquare$) was based on the preoperative (PO) image to construct the JCS $A_{PO}$.

When the FSU is at rest in this configuration, the external wrench applied between the vertebrae in frame $A_{PO}$ is:

$$_{A_{PO}}\vec {w}(t_{IO}) \equiv 0$$

We express a general wrench as a combination of force and torque vectors around the origin of frame $A_i$:

$$\begin{aligned} {}_{A_i}\vec {w}(t) = \begin{pmatrix} \vec {F}(t) \\ \vec {\tau }(t) \end{pmatrix} = \begin{pmatrix} F_x(t), F_y(t), F_z(t), \tau _x(t), \tau _y(t), \tau _z(t) \end{pmatrix}^T \end{aligned}$$

To displace the FSU from this equilibrium, an external wrench must be applied for $t > t_{IO}$:

$$_{A_{PO}}\vec {w}(t > t_{IO}) \not \equiv 0$$

The resulting displacement is described by $\vec {\theta }_{PO}(t)$. Relating a wrench applied in frame *A* to the resulting transformation from frame *B* to *A* yields:

$$\begin{aligned} {}_{A}\vec {w}(t) = f({}_{B}T_{A}(t)) \end{aligned}$$

Expressing displacement relative to $A_{PO}$ introduces interpretation challenges:

i) Achieved spinal loading from a deflected $\displaystyle V_{2}$ would result in an offset term in the function $\displaystyle f$. While correcting for this offset by, e.g., $\displaystyle _{A_{PO}}\vec {w}(t) = f(\vec {\theta }_{PO}(t) - c)$ would be possible, it could be confusing. ii) Single-DoF motions are based on the preoperative orientation, not on visible IO anatomy. That means surgeons applying single-axis motion to measure the resulting wrench would have to command such motion relative to the axis definitions of the preoperative image and map safe motion limits themselves from their intraoperative view to the preoperative space, while looking at the intraoperative vertebrae orientation. iii) Wrench application along PO axes may lead to unintended translations and/or rotations of other axes due to misalignment with the intraoperative state that could be harmful for the patient.(Fig. 3e).

To address these limitations, a new reference frame $A_{IO}$ is defined at $t_{IO}$. A virtual 6-DoF joint $\blacktriangle$ is generated using ${}_{V_2}\tilde{T}_{V_1}(t_{IO})$ (Fig. 3f), enabling motion description using intraoperative-based coordinates:

$$\vec {\theta }_{IO}(t) = \begin{pmatrix} \theta _{IO,x}(t), \theta _{IO,y}(t), \theta _{IO,z}(t); \\ \theta _{IO,\alpha }(t), \theta _{IO,\beta }(t), \theta _{IO,\gamma }(t) \end{pmatrix}^T$$

With this IO FSU reference, spinal loading and resulting displacement are defined by:

$$\begin{aligned} {}_{A_{IO}}\vec {w}(t \ge t_{IO}) = f(\vec {\theta }_{IO}(t \ge t_{IO})) \end{aligned}$$

where $${\vec {\theta }_{IO}(t_{IO}) \equiv 0}$$ and $${}_{A_{IO}}\vec {w}(t_{IO}) \equiv 0$$.

### Case Study: Vertebrae Shift

We obtained intraoperative transform $_{V_1}\tilde{T}_{V_2}(t=t_{IO})$ by fitting vertebra to vertebra meshes using the Iterative Closest Point (ICP) algorithm from the MATLAB r2023b Computer Vision Toolbox (MathWorks, Natick, MA, USA). The mesh fit overlapped preoperatively segmented, coordinate-aligned vertebrae on intraoperatively segmented scans of the corresponding vertebra in the intraoperative state. We chose this approach because the C-Arm-produced scans were fractured and had low-resolution quality. ICP parameters were manually tuned to produce fits that appeared reasonable upon visual inspection of the alignment. The following parameters for FSU T9-T10/FSU T10-T11 were used: Inliner ratio: 0.8, iteration: 500, tolerance interval:[0.01 mm- 0.1 mm].We performed the ICP algorithm three times for each FSU.

To quantitatively assess vertebrae displacement, we chose the following dissimilarity metrics:

- Translation: Calculated as the Euclidean distance between coordinate system origins $V_1$ and $V_2$.
  $$\Vert \Delta \vec {r}\Vert = \Vert {_{V_2}\vec {r}_{O_{V_1}}}(t_{IO}) - {_{V_2}\vec {r}_{O_{V_1}}}(t_{PO})\Vert$$
- Rotation: The relative rotation error is computed as the geodesic distance between the rotation matrices.
  $$\Delta R = \arccos \left( \frac{\text {trace}\{ _{V_2}R_{V_1}(t_{PO}) _{V_2}R_{V_1}^T(t_{IO})\} - 1}{2}\right)$$

### Dissimilarity Study: Virtual Motion and Required Wrench Capability

#### Virtual FSU Motion

To test **Hypothesis 1** and **Hypothesis 2**, we performed virtual FSU motion for discrete single-axis angles of ± $5^{\circ }$, $10^{\circ }$, and $15^{\circ }$. We chose these bending angles because they reflect typical SLS-induced bending motions found in literature (e.g., [2]). Additionally, we chose to performed single-axis translations of ± 1 mm, 2 mm, and 3 mm. In addition, we applied single-axis translations of ±1, 2, and 3 mm, chosen to approximate the physiological range of motion. We analyzed the resulting poses by applying these virtual motions in four different conditions, manipulating the rigid body tree configurations with $\vec {\theta }(t)$. We applied them for preoperatively or intraoperatively defined JCS $A_{PO}$ or $A_{IO}$. We considered both the *Single Parent* and *Double Parents* definitions of the intervertebral reference axis directions. We evaluated dissimilarities between resulting FSU poses (expressed with the transform ${\displaystyle _{V2}\tilde{T}_{V_{1}{}}( t>t_{IO})}$) from the different reference time points $A_{PO}$ and $A_{IO}$ or the different axis directions *Single Parent* and *Double Parents* with the metrics Euclidean distance and rotation error (described in Sect. 2.4).

#### Required Wrench Capability

**Fig. 4:**
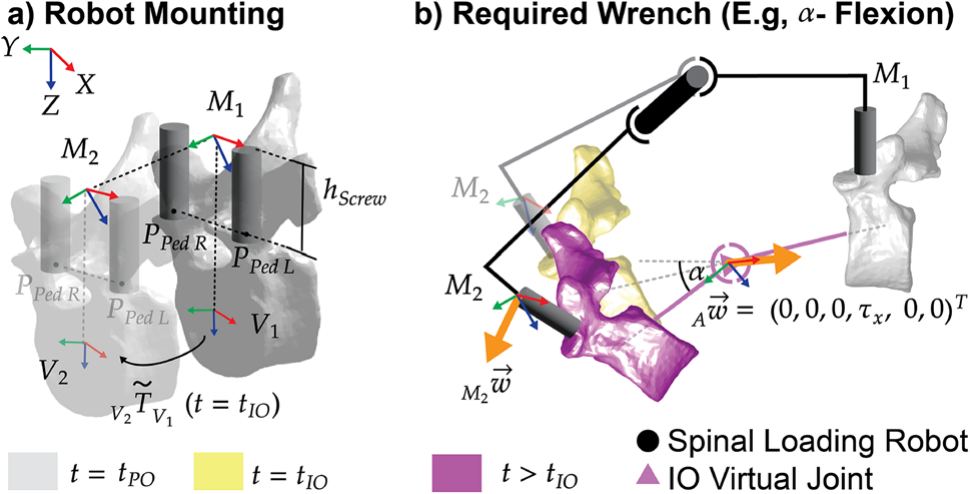
**a**) FSU annotation of pedicle screw entry positions and pedicle screw-mounted spinal loading wrench loci $M_1$ and $M_2$. $h_{screw}$ is the minimum height offset that allows for device mounting points $M_1$ and $M_2$, and it depends on the screw dimensions. **b**) Schematic spinal loading displacement from intraoperative FSU State using flexion angle $\alpha$ (in x-axis) as illustration. A single-axis pure bending wrench (orange arrow) is applied at intervertebral joint A as $_{A}\vec {w}$ and is calculated at $M_2$ using the adjoint transformation matrix for wrench analysis in a spinal loading device.

To test **Hypothesis 3**, we evaluated the required wrench capability of a spinal loading device when the FSU is in a virtual motion pose. We chose to analyze the required wrench at Mounting Point $M_2$ that acted as the end effector of a spinal loading device (Fig. 4a). We subjected the produced FSU configuration to pure translation and bending conditions, where a single-axis force or torque was applied at the JCS along the direction of displacement. We chose discrete torque values of ±5, 7.5, and 10 Nm for $\tau _{x}$, $\tau _{y}$, and $\tau _{z}$, as they reflect typical SLS-induced torques reported in the literature (e.g., [2]). For force values, we selected ±5, 10, and 15 N for $F_{x}$, $F_{y}$, and $F_{z}$ to represent the relevant interaction load ranges. This choice is supported by animal vertebra palpation instrumentation studies from Zoller et al., who measured probe-vertebra palpation forces of up to approximately 7.78 N. [30] We calculated the required wrench capability for the virtual motions stated in section 2.4.1.

We extended the rigid body tree model by wrench application points constructed using pedicle screw entry positions and screw height (Fig. 4a). Entry positions were annotated by a physician using Brainlab (Brainlab AG, Munich, Germany) spinal surgery planning software, based on the preoperative CT scan. Mounting points $M_{1}$ and $M_{2}$ were defined as the vertical projection in $\vec {e}_{x_{V_{1}}}$ and $\vec {e}_{x_{V_{2}}}$ from the midpoint of vector $\overline{P_{Ped R}P_{Ped L}}$ of the respective vertebra.

$$\begin{aligned} {_{V_i}\vec {r}_{M_{i}}} ={_{V_i}\vec {r}_{P_{PedR,V_i}}} + \frac{1}{2}\overline{P_{PedR,V_i}P_{PedL,V_i}} + {_{V_i}(0,-h_{screw},0)^T} \end{aligned}$$

We assumed a wrench-delivering measurement device to be mounted on a rod bridging the two installed pedicle screws on each vertebra. We chose a minimal offset height by the used VERSE pedicle screw of 25 mm. We defined the root coordinates for mounting points $M_1$ and $M_2$ as $f_{Coord}(\vec {r}_{O_{M_1}},\vec {c}_{M_1},\vec {d}_{M_1})$ and $f_{Coord}(\vec {r}_{O_{M_2}},-\vec {c_{M_1}}, \vec {d}_{M_2},)$ with $\vec {c}_{M_1} = \frac{1}{2}\overline{M_{1}M_{2}}$ and $\vec {d}_{Vi} = \frac{1}{2}\overline{P_{Ped R, V_i}P_{Ped L, V_i}}$ of the respective vertebra. The required wrench $_{M_2}\vec {w}$ (Fig. 4b) provided by a robotic measurement device expressed in its frame $M_2$ to realize the desired wrench $_{A}\vec {w}$ around the intervertebral reference *A* was obtained by left multiplication of the adjoint transformation matrix ${_{M_2}T_{A}}$:

$$\begin{aligned} {_{M_2}\vec {w}} = \text {Ad}({_{M_2}T_{A}})^\top \, _{A}\vec {w}_{A}. \end{aligned}$$

where $\text {Ad}({_{M_2}T_{A}})$ was defined as:

$$\begin{aligned} \text {Ad}(_{M_2}T_A) = \begin{bmatrix} {_{M_2}R_A} & \hat{p}({_{B}\vec {r}_{O_A}}) \,* {_{M_2}R_A} \\ 0 & {_{M_2}R_A} \end{bmatrix} \end{aligned}$$

$\hat{p}$ denoted the skew-symmetric matrix.

Forces acting on the JCS translate to a torque due to the lever arm of the JCS to the mounting point $M_2$. Similarly, torques acting on the JCS translate to a force due to the lever arm of JCS to the mounting point $M_2$.

Dissimilarity analysis between wrenches $_{M_2}\vec {w}$ was assessed separately for force and torque components using the Euclidean norm:

- Force: Calculated as the Euclidean norm between force dissimilarity.
  $$\quad \Vert \Delta \vec {F}\Vert = \Vert \vec {F}(t_{IO}) - \vec {F}(t_{PO})\Vert$$
- Torque: Calculated as the Euclidean norm between torque dissimilarity.
  $$\quad \Vert \Delta \vec {\tau }\Vert = \Vert \vec {\tau }(t_{IO}) - \vec {\tau }(t_{PO})\Vert$$

## Results

### Case Study: Vertebrae Shift

**Fig. 5:**
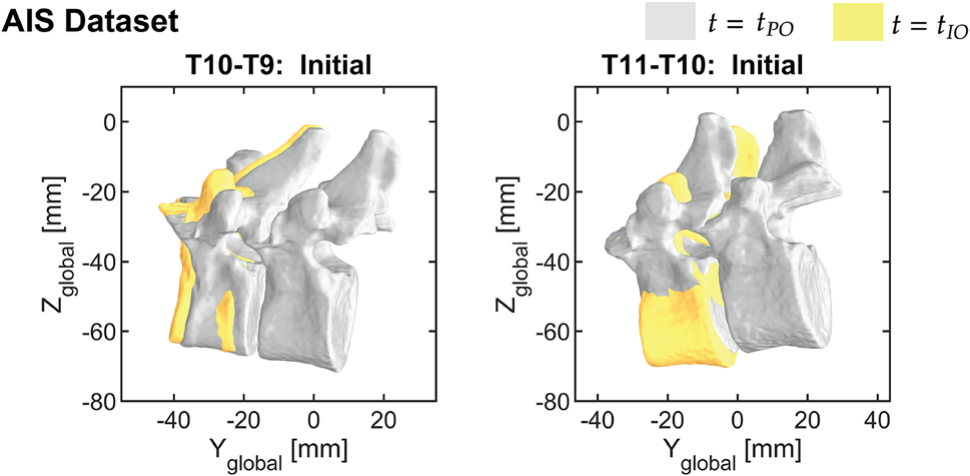
Overlay of the locations of the second vertebra between preoperative (gray) to intraoperative (yellow) FSU pose for FSU T9-T10 and T10-T11.

The Euclidean distance and geodesic distance between the locations of the second vertebra in preoperative and intraoperative FSU pose resulted in up to 3.21 mm and $28.35^{\circ }$, respectively. (Table 1). Using the same ICP Fit parameters resulted in a variability of $_{V_2}\tilde{T}_{V_1} (t_{IO})$ and thus the dissimilarity metrics. We chose to keep $_{V_2}\tilde{T}_{V_1} (t_{IO})$ from the ICP fit 1 and keep it constant for the following calculations. The produced vertebral shift of FSU T9-T10 and T10-T11 are displayed in Fig. 5.

**Table 1:** Dissimilarity for FSUs T9-T10 and T10-T11 between preoperative to intraoperative FSU pose. ICP Fit #1 (**bold**) is used for further calculations

FSU	T9-T10			T10-T11		
ICP Fit #	$\Vert \Delta \vec {r}\Vert \,[{\mathrm{mm}}]$	$\Delta R [^{\circ }]$	$\frac{\Vert \Delta \vec {r}\Vert }{\Vert \vec {r}_{IO}\Vert }$ [$\%$]	$\Vert \Delta \vec {r}\Vert$[mm]	$\Delta R [^{\circ }]$	$\frac{\Vert \Delta \vec {r}\Vert }{\Vert \vec {r}_{IO}\Vert }$ [$\%$]
**1**	**3**.**02**	**15**.**26**	**10**.**91**	**3**.**19**	**28**.**34**	**11**.**88**
2	2.98	15.20	10.79	3.19	28.34	11.86
3	3.00	15.18	10.88	3.21	28.35	11.96

### Dissimilarity Study: Virtual Motion and Required Wrench Capability

#### Single Parent

The Euclidean distance between the resulting locations of the second vertebra, when applying the same motion to either preoperatively or intraoperatively defined *Single Parent* coordinates, resulted in 0.42 mm for FSU T10-T11 when applying a pure axial rotation of $\delta \beta = 15^{\circ }$ (Table 2). We measured small dissimilarities in Euclidean distance of up to 0.01$\%$ between the resulting locations of the second vertebra between positive and negative FSU virtual motion. As an example, we chose to show this effect for lateral bending ($\delta \gamma = \pm 15$ mm, FSU T9-T10) where the difference is largest (Table 2). Complete Tables are found in the supplementary.

**Fig. 6:**
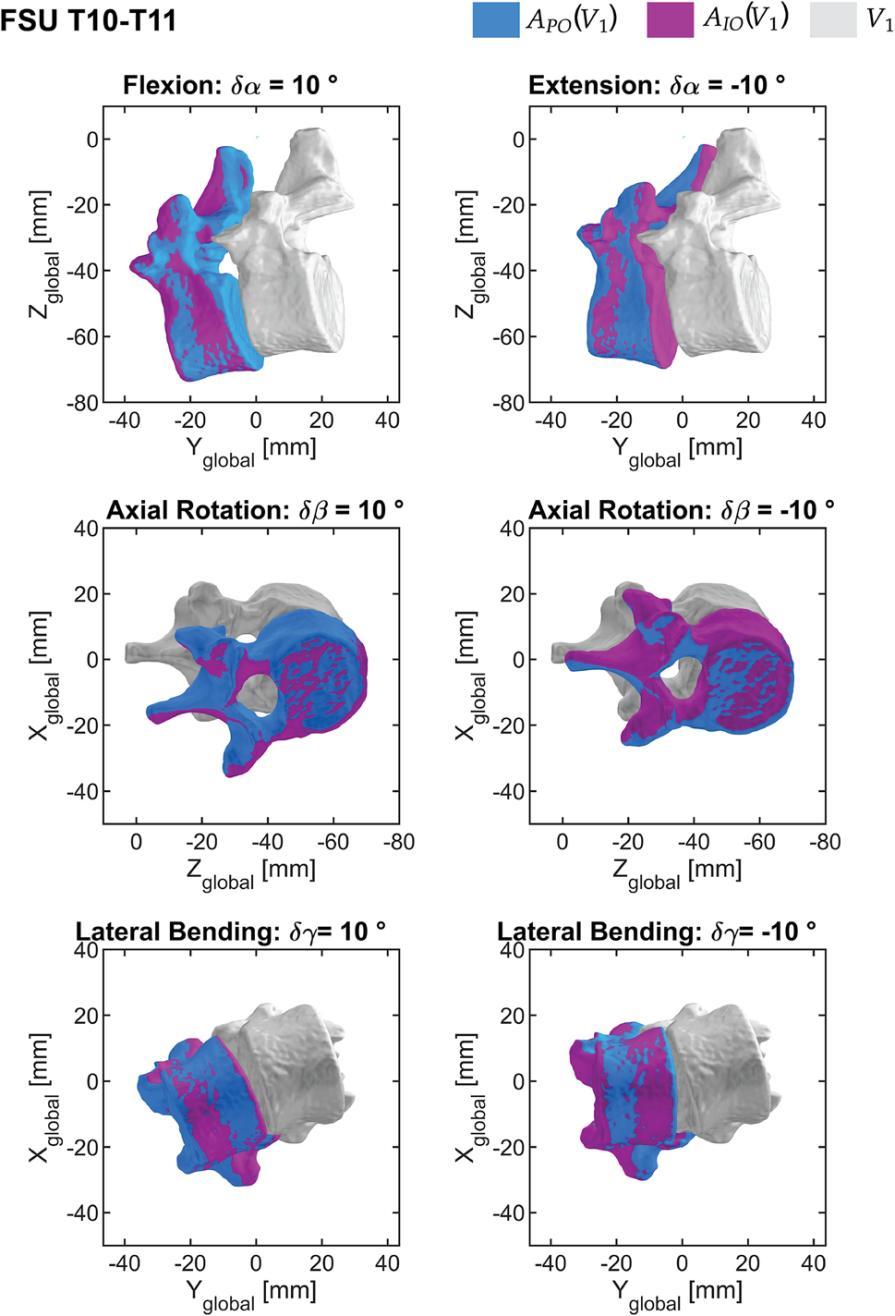
Overlay of the resulting locations of the second vertebra when applying the same virtual bending motion to either preoperative (blue) or intraoperative (purple) *Single Parent*-defined coordinates for FSU T10-T11.

We chose to visually display the Euclidean distances for $10^{\circ }$ bending for illustrative purposes (Fig. 6). We provided similar plots for virtual motion for $10^{\circ }$ bending for FSU T9-T10 and T10-T11 in the supplementary. Applying pure translation virtual motions to the same FSU axis directions in *Single Parent* leads to the same location of the second vertebra when applying the same motion to either preoperatively or intraoperatively defined *Single Parent* coordinates and is therefore not displayed in Table 2.

The torque dissimilarity that arises in the pure translation virtual motion resulted in up to 1.12 mNm in the compression/decompression direction at $\pm 10$ Nm (Table 3). Similar to Table 2, we chose to show the largest difference in torque dissimilarity of up to 0.01$\%$ depending on the directions for Translation AP/PA. Applying pure bending virtual motions to the same FSU axis direction in *Single Parent* leads to the same force and torque dissimilarities when applying the same motion to either preoperatively or intraoperatively defined *Single Parent* coordinates. We therefore do not display the correspondig force and torque dissimilarities in Table  3.

**Table 2:** Dissimilarities between the resulting locations of the second vertebra when applying the same motion to either preoperative or intraoperative *Single Parent*-defined coordinates for FSU T9-T10 and FSU T10-T11

Flexion/Extension						
FSU	T9-T10			T10-T11		
$\delta \alpha [^{\circ }]$	$\Vert \Delta \vec {r}\Vert \,[{\mathrm{mm}}]$	$\Delta R [^{\circ }]$	$\frac{\Vert \Delta \vec {r}\Vert }{\Vert \vec {r}_{IO}\Vert }$ [$\%$]	$\Vert \Delta \vec {r}\Vert \,[{\mathrm{mm}}]$	$\Delta R [^{\circ }]$	$\frac{\Vert \Delta \vec {r}\Vert }{\Vert \vec {r}_{IO}\Vert }$ [$\%$]
± 5	0.13	0.00	0.46	0.13	0.00	0.48
± 10	0.25	0.00	0.92	0.26	0.00	0.97
± 15	0.38	0.00	1.39	0.39	0.00	1.45

Axial Rotation R/L						
FSU	T9-T10			T10-T11		
$\delta \beta [^{\circ }]$	$\Vert \Delta \vec {r}\Vert \,[{\mathrm{mm}}]$	$\Delta R [^{\circ }]$	$\frac{\Vert \Delta \vec {r}\Vert }{\Vert \vec {r}_{IO}\Vert }$ [$\%$]	$\Vert \Delta \vec {r}\Vert \,[{\mathrm{mm}}]$	$\Delta R [^{\circ }]$	$\frac{\Vert \Delta \vec {r}\Vert }{\Vert \vec {r}_{IO}\Vert }$ [$\%$]
± 5	0.03	0.00	0.11	0.14	0.00	0.52
± 10	0.06	0.00	0.22	0.28	0.00	1.04
± 15	0.09	0.00	0.33	0.42	0.00	1.55

Lateral Bending R/L						
FSU	T9-T10			T10-T11		
$\delta \gamma [^{\circ }]$	$\Vert \Delta \vec {r}\Vert \,[{\mathrm{mm}}]$	$\Delta R [^{\circ }]$	$\frac{\Vert \Delta \vec {r}\Vert }{\Vert \vec {r}_{IO}\Vert }$ [$\%$]	$\Vert \Delta \vec {r}\Vert \,[{\mathrm{mm}}]$	$\Delta R [^{\circ }]$	$\frac{\Vert \Delta \vec {r}\Vert }{\Vert \vec {r}_{IO}\Vert }$ [$\%$]
± 5	0.13	0.00	0.47	0.06	0.00	0.24
± 10	0.26	0.00	0.95	0.13	0.00	0.49
− 15	0.39	0.00	1.43	0.19	0.00	0.74
$+$ 15	0.39	0.00	1.42	0.19	0.00	0.74

**Table 3:** Dissimilarities between the required wrench capability (force and torque) when applying the same motion under same single axis loading to either preoperative and intraoperative *Single Parent*-defined coordinates for FSU T9-T10 and FSU T10-T11

Wrench: Translation M/L @ $\Delta F_x = \pm 7.5$ N								
FSU	T9-T10				T10-T11			
$\delta x$ [mm]	$\Vert \Delta \vec {F}\Vert$ [mN]	$\Vert \Delta \vec {\tau }\Vert$ [mNm]	$\frac{\Vert \Delta \vec {F}\Vert }{\Vert \vec {F}_{IO}\Vert }$ [$\%$]	$\frac{\Vert \Delta \vec {\tau }\Vert }{\Vert \vec {\tau }_{IO}\Vert }$ [$\%$]	$\Vert \Delta \vec {F}\Vert$[mN]	$\Vert \Delta \vec {\tau }\Vert$[mNm]	$\frac{\Vert \Delta \vec {F}\Vert }{\Vert \vec {F}_{IO}\Vert }$ [$\%$]	$\frac{\Vert \Delta \vec {\tau }\Vert }{\Vert \vec {\tau }_{IO}\Vert }$ [$\%$]
± 2	0.00	1.06	0.00	2.64	0.00	1.08	0.00	2.60

Wrench: Compression/Decompression @ $\Delta F_y = \pm 7.5$ N								
FSU	T9-T10				T10-T11			
$\delta y$ [mm]	$\Vert \Delta \vec {F}\Vert$ [mN]	$\Vert \Delta \vec {\tau }\Vert$ [mNm]	$\frac{\Vert \Delta \vec {F}\Vert }{\Vert \vec {F}_{IO}\Vert }$ [$\%$]	$\frac{\Vert \Delta \vec {\tau }\Vert }{\Vert \vec {\tau }_{IO}\Vert }$ [$\%$]	$\Vert \Delta \vec {F}\Vert$ [mN]	$\Vert \Delta \vec {\tau }\Vert$ [mNm]	$\frac{\Vert \Delta \vec {F}\Vert }{\Vert \vec {F}_{IO}\Vert }$ [$\%$]	$\frac{\Vert \Delta \vec {\tau }\Vert }{\Vert \vec {\tau }_{IO}\Vert }$ [$\%$]
± 2	0.00	0.25	0.00	0.67	0.00	1.12	0.00	3.10

Wrench: Translation AP/PA @ $\Delta F_z = \pm 7.5$ N								
FSU	T9-T10				T10-T11			
$\delta z$ [mm]	$\Vert \Delta \vec {F}\Vert$ [mN]	$\Vert \Delta \vec {\tau }\Vert$ [mNm]	$\frac{\Vert \Delta \vec {F}\Vert }{\Vert \vec {F}_{IO}\Vert }$ [$\%$]	$\frac{\Vert \Delta \vec {\tau }\Vert }{\Vert \vec {\tau }_{IO}\Vert }$ [$\%$]	$\Vert \Delta \vec {F}\Vert$ [mN]	$\Vert \Delta \vec {\tau }\Vert$ [mNm]	$\frac{\Vert \Delta \vec {F}\Vert }{\Vert \vec {F}_{IO}\Vert }$ [$\%$]	$\frac{\Vert \Delta \vec {\tau }\Vert }{\Vert \vec {\tau }_{IO}\Vert }$ [$\%$]
$+$ 2	0.00	1.11	0.00	7.25	0.00	0.36	0.00	4.21
− 2	0.00	1.11	0.00	7.25	0.00	0.36	0.00	4.22

#### Double Parent

The Euclidean distance between the resulting locations of the second vertebra, when applying the same motion to either preoperatively or intraoperatively defined *Double Parent* coordinates, resulted to 0.38 mm when applying a pure flexion of $\delta \alpha = 10^{\circ }$ (Table 4). A geodesic distance of up to $24.18^{\circ }$ in the same direction is obtained. Additionally, we recorded small differences up to 0.05$\%$ in Euclidean distances between the resulting locations of the second vertebra between positive and negative FSU virtual motion. As an example, we showed this effect for lateral bending, where the percentage Euclidean distance is the largest (Table 4). In this section, we have chosen to display virtual motions of $\pm 2$ mm translational and $\pm 10^{\circ }$ for bending directions, as these displacements are representative of the entire dataset. Complete table and visually displayed FSU motions ($\pm 10^{\circ }$, $\pm 2$ mm) (similar to Fig. 6 are found in the supplementary.

The force dissimilarity in pure translation virtual motion resulted in 0.94 N fortranslation AP of $\delta z = 2$ mm.(Table 5)). We obtained torque dissimilarity in pure bending virtual motions of up to 0.95 Nm for lateral bending L of $\delta \gamma = 10^{\circ }$. (Table 5)). Applying pure bending virtual motions to the same FSU axis direction in *Double Parent* leads to the same force dissimilarities and are therefore not displayed in Table 5. We choose to show force and torque metrics for $\Delta F_i = \pm 7.5$ N and $\Delta \tau _i = \pm 7.5$ Nm for conciseness. The complete table is available in the supplementary. Additionally, we recorded force differences of up to 0.01$\%$ and torque dissimilarities of up to 0.09$\%$ between the resulting wrench input between positive and negative FSU virtual motion. For example, we illustrate this effect for lateral bending, where it is the largest (Table 5). The complete table and visually displayed FSU motions are found in the supplementary.

**Table 4:** Dissimilarities between the resulting locations of the second vertebra when applying the same motion to either preoperative or intraoperative *Double Parent*-defined coordinates for FSU T9-T10 and FSU T10-T11

Translation M/L						
FSU	T9-T10			T10-T11		
$\delta x\,[{\mathrm{mm}}]$	$\Vert \Delta \vec {r}\Vert\,[{\mathrm{mm}}]$	$\Delta R [^{\circ }]$	$\frac{\Vert \Delta \vec {r}\Vert }{\Vert \vec {r}_{IO}\Vert }$ $\mathrm{[\%]}$	$\Vert \Delta \vec {r}\Vert\,[{\mathrm{mm}}]$	$\Delta R [^{\circ }]$	$\frac{\Vert \Delta \vec {r}\Vert }{\Vert \vec {r}_{IO}\Vert }$ $\mathrm{[\%]}$
+2	0.17	0.00	0.62	0.24	0.00	0.90

Compression/Decompression						
FSU	T9-T10			T10-T11		
$\delta y\,[{\mathrm{mm}}]$	$\Vert \Delta \vec {r}\Vert\,[{\mathrm{mm}}]$	$\Delta R [^{\circ }]$	$\frac{\Vert \Delta \vec {r}\Vert }{\Vert \vec {r}_{IO}\Vert }$ $\mathrm{[\%]}$	$\Vert \Delta \vec {r}\Vert\,[{\mathrm{mm}}]$	$\Delta R [^{\circ }]$	$\frac{\Vert \Delta \vec {r}\Vert }{\Vert \vec {r}_{IO}\Vert }$ $\mathrm{[\%]}$
+2	0.07	0.00	0.27	0.08	0.00	0.26

Translation AP/PA						
FSU	T9-T10			T10-T11		
$\delta z\,[{\mathrm{mm}}]$	$\Vert \Delta \vec {r}\Vert\,[{\mathrm{mm}}]$	$\Delta R [^{\circ }]$	$\frac{\Vert \Delta \vec {r}\Vert }{\Vert \vec {r}_{IO}\Vert }$ $\mathrm{[\%]}$	$\Vert \Delta \vec {r}\Vert\,[{\mathrm{mm}}]$	$\Delta R [^{\circ }]$	$\frac{\Vert \Delta \vec {r}\Vert }{\Vert \vec {r}_{IO}\Vert }$ $\mathrm{[\%]}$
+2	0.16	0.00	0.57	0.27	0.00	0.94

Flexion/Extension						
FSU	T9-T10			T10-T11		
$\delta \alpha [^{\circ }]$	$\Vert \Delta \vec {r}\Vert\,[{\mathrm{mm}}]$	$\Delta R [^{\circ }]$	$\frac{\Vert \Delta \vec {r}\Vert }{\Vert \vec {r}_{IO}\Vert }$ $\mathrm{[\%]}$	$\Vert \Delta \vec {r}\Vert\,[{\mathrm{mm}}]$	$\Delta R [^{\circ }]$	$\frac{\Vert \Delta \vec {r}\Vert }{\Vert \vec {r}_{IO}\Vert }$ $\mathrm{[\%]}$
+10	0.32	11.50	1.19	0.38	24.18	1.38

Axial Rotation R/L						
FSU	T9-T10			T10-T11		
$\delta \beta [^{\circ }]$	$\Vert \Delta \vec {r}\Vert\,[{\mathrm{mm}}]$	$\Delta R [^{\circ }]$	$\frac{\Vert \Delta \vec {r}\Vert }{\Vert \vec {r}_{IO}\Vert }$ $\mathrm{[\%]}$	$\Vert \Delta \vec {r}\Vert\,[{\mathrm{mm}}]$	$\Delta R [^{\circ }]$	$\frac{\Vert \Delta \vec {r}\Vert }{\Vert \vec {r}_{IO}\Vert }$ $\mathrm{[\%]}$
+10	0.16	11.48	0.56	0.20	24.16	0.72

Lateral Bending R/L						
FSU	T9-T10			T10-T11		
$\delta \gamma [^{\circ }]$	$\Vert \Delta \vec {r}\Vert\,[{\mathrm{mm}}]$	$\Delta R [^{\circ }]$	$\frac{\Vert \Delta \vec {r}\Vert }{\Vert \vec {r}_{IO}\Vert }$ $\mathrm{[\%]}$	$\Vert \Delta \vec {r}\Vert\,[{\mathrm{mm}}]$	$\Delta R [^{\circ }]$	$\frac{\Vert \Delta \vec {r}\Vert }{\Vert \vec {r}_{IO}\Vert }$ $\mathrm{[\%]}$
+10	0.32	11.50	1.18	0.32	24.19	1.26
-10	0.32	11.50	1.17	0.34	24.19	1.21

**Table 5:** Dissimilarities between the required wrench capability (force and torque) when applying the same motion under same single axis loading to either preoperative and intraoperative *Double Parent*-defined coordinates for FSU T9-T10 and FSU T10-T11

Wrench: Translation M/L @ $\Delta F_x = \pm 7.5$ N								
FSU	T9-T10				T10-T11			
$\delta x\,[{\mathrm{mm}}]$	$\Vert \Delta \vec {F}\Vert\mathrm{\,[N]}$	$\Vert \Delta \vec {\tau }\Vert\mathrm{\,[Nm]}$	$\frac{\Vert \Delta \vec {F}\Vert }{\Vert \vec {F}_{IO}\Vert }$ $\mathrm{[\%]}$	$\frac{\Vert \Delta \vec {\tau }\Vert }{\Vert \vec {\tau }_{IO}\Vert }$ $\mathrm{[\%]}$	$\Vert \Delta \vec {F}\Vert\mathrm{\,[N]}$	$\Vert \Delta \vec {\tau }\Vert\mathrm{\,[Nm]}$	$\frac{\Vert \Delta \vec {F}\Vert }{\Vert \vec {F}_{IO}\Vert }$ $\mathrm{[\%]}$	$\frac{\Vert \Delta \vec {\tau }\Vert }{\Vert \vec {\tau }_{IO}\Vert }$ $\mathrm{[\%]}$
+2	0.63	0.01	8.65	2.44	0.89	0.02	12.60	4.62

Wrench: Compression/Decompression @ $\Delta F_y = \pm 7.5$ N								
FSU	T9-T10				T10-T11			
$\delta y\,[{\mathrm{mm}}]$	$\Vert \Delta \vec {F}\Vert\mathrm{\,[N]}$	$\Vert \Delta \vec {\tau }\Vert\mathrm{\,[Nm]}$	$\frac{\Vert \Delta \vec {F}\Vert }{\Vert \vec {F}_{IO}\Vert }$ $\mathrm{[\%]}$	$\frac{\Vert \Delta \vec {\tau }\Vert }{\Vert \vec {\tau }_{IO}\Vert }$ $\mathrm{[\%]}$	$\Vert \Delta \vec {F}\Vert\mathrm{\,[N]}$	$\Vert \Delta \vec {\tau }\Vert\mathrm{\,[Nm]}$	$\frac{\Vert \Delta \vec {F}\Vert }{\Vert \vec {F}_{IO}\Vert }$ $\mathrm{[\%]}$	$\frac{\Vert \Delta \vec {\tau }\Vert }{\Vert \vec {\tau }_{IO}\Vert }$ $\mathrm{[\%]}$
+2	0.26	0.02	3.41	4.19	0.28	0.01	3.74	2.03

Wrench: Translation AP/PA @ $\Delta F_y = \pm 7.5$ N								
FSU	T9-T10				T10-T11			
$\delta z\,[{\mathrm{mm}}]$	$\Vert \Delta \vec {F}\Vert\mathrm{\,[N]}$	$\Vert \Delta \vec {\tau }\Vert\mathrm{\,[Nm]}$	$\frac{\Vert \Delta \vec {F}\Vert }{\Vert \vec {F}_{IO}\Vert }$ $\mathrm{[\%]}$	$\frac{\Vert \Delta \vec {\tau }\Vert }{\Vert \vec {\tau }_{IO}\Vert }$ $\mathrm{[\%]}$	$\Vert \Delta \vec {F}\Vert\mathrm{\,[N]}$	$\Vert \Delta \vec {\tau }\Vert\mathrm{\,[Nm]}$	$\frac{\Vert \Delta \vec {F}\Vert }{\Vert \vec {F}_{IO}\Vert }$ $\mathrm{[\%]}$	$\frac{\Vert \Delta \vec {\tau }\Vert }{\Vert \vec {\tau }_{IO}\Vert }$ $\mathrm{[\%]}$
+2	0.58	0.04	7.93	23.81	0.94	0.05	13.43	34.97

Wrench: Flexion/Extension @ $\Delta \tau _x = \pm 7.5$ Nm								
FSU	T9-T10				T10-T11			
$\delta \alpha [^{\circ }]$	$\Vert \Delta \vec {F}\Vert\mathrm{\,[N]}$	$\Vert \Delta \vec {\tau }\Vert\mathrm{\,[Nm]}$	$\frac{\Vert \Delta \vec {F}\Vert }{\Vert \vec {F}_{IO}\Vert }$ $\mathrm{[\%]}$	$\frac{\Vert \Delta \vec {\tau }\Vert }{\Vert \vec {\tau }_{IO}\Vert }$ $\mathrm{[\%]}$	$\Vert \Delta \vec {F}\Vert\mathrm{\,[N]}$	$\Vert \Delta \vec {\tau }\Vert\mathrm{\,[Nm]}$	$\frac{\Vert \Delta \vec {F}\Vert }{\Vert \vec {F}_{IO}\Vert }$ $\mathrm{[\%]}$	$\frac{\Vert \Delta \vec {\tau }\Vert }{\Vert \vec {\tau }_{IO}\Vert }$ $\mathrm{[\%]}$
+10	0.00	0.63	NaN	8.66	0.00	0.89	NaN	12.61

Wrench: Axial Rotation @ $\Delta \tau _y = \pm 7.5$ Nm								
FSU	T9-T10				T10-T11			
$\delta \beta [^{\circ }]$	$\Vert \Delta \vec {F}\Vert\mathrm{\,[N]}$	$\Vert \Delta \vec {\tau }\Vert\mathrm{\,[Nm]}$	$\frac{\Vert \Delta \vec {F}\Vert }{\Vert \vec {F}_{IO}\Vert }$ $\mathrm{[\%]}$	$\frac{\Vert \Delta \vec {\tau }\Vert }{\Vert \vec {\tau }_{IO}\Vert }$ $\mathrm{[\%]}$	$\Vert \Delta \vec {F}\Vert\mathrm{\,[N]}$	$\Vert \Delta \vec {\tau }\Vert\mathrm{\,[Nm]}$	$\frac{\Vert \Delta \vec {F}\Vert }{\Vert \vec {F}_{IO}\Vert }$ $\mathrm{[\%]}$	$\frac{\Vert \Delta \vec {\tau }\Vert }{\Vert \vec {\tau }_{IO}\Vert }$ $\mathrm{[\%]}$
+10	0.00	0.26	NaN	3.41	0.00	0.28	NaN	3.74

Wrench: Lateral Bending @ $\Delta \tau _z = \pm 7.5$ Nm								
FSU	T9-T10				T10-T11			
$\delta \beta [^{\circ }]$	$\Vert \Delta \vec {F}\Vert\mathrm{\,[N]}$	$\Vert \Delta \vec {\tau }\Vert\mathrm{\,[Nm]}$	$\frac{\Vert \Delta \vec {F}\Vert }{\Vert \vec {F}_{IO}\Vert }$ $\mathrm{[\%]}$	$\frac{\Vert \Delta \vec {\tau }\Vert }{\Vert \vec {\tau }_{IO}\Vert }$ $\mathrm{[\%]}$	$\Vert \Delta \vec {F}\Vert\mathrm{\,[N]}$	$\Vert \Delta \vec {\tau }\Vert\mathrm{\,[Nm]}$	$\frac{\Vert \Delta \vec {F}\Vert }{\Vert \vec {F}_{IO}\Vert }$ $\mathrm{[\%]}$	$\frac{\Vert \Delta \vec {\tau }\Vert }{\Vert \vec {\tau }_{IO}\Vert }$ $\mathrm{[\%]}$
+10	0.00	0.58	NaN	7.93	0.00	0.95	NaN	13.47
-10	0.00	0.58	NaN	7.92	0.00	0.94	NaN	13.38

## Discussion

### Case Study: Vertebrae Shift

We applied our FSU motion parametrization to determine vertebral 3-dimensional shifts from preoperative to intraoperative FSU poses for FSU T9-T10 and T10-T11 of one AIS patient. We measured relative coordinate position and angular displacement of the second vertebra of 3.00$-$3.21 mm and $15.18-28.34^{\circ }$ from preoperative to interoperative status of the FSU T9-T10 and T10-T11.

**Limitation of intraoperative imaging data**: Our approach relied on the segmentation of a high-resolution image technique (e.g, low-dose CT Scan) to create an aligned and detailed mesh fitted to the segmentation of a noisy and fractured mesh produced with a low-resolution imaging technique. The reduced resolution of the C-arm scan impacted the ICP fit performance. This became evident as the ICP fit algorithm yielded varying locations of the cranial vertebrae with the same input parameters, as shown in Table 1. This effect limits the study’s specific outcome as the ICP fit was a precondition for further analysis, such as the motion parameterization, which directly depends on the reference systems of the vertebrae used. However, the specific imaging modality, registration, or fitting algorithms can be exchanged. We assume that improved intraoperative imaging could enhance the robustness of the vertebra reference system and, thereby, lead to a more accurate definition of the intervertebral joint and motion studies.

**Limitation of landmark amount and accuracy**: Our approach to obtaining local coordinate systems was based on a modified six-point method from André et al. [23]. This method relies solely on vertebral body and pedicle landmarks, but not on posterior elements. Posterior elements may be deformed in AIS pathology and would therefore be less reliable. The current implementation still relied heavily on manual landmark selection and was influenced by the mesh density of the CT-segmentation. Here, vertebra registration approaches with more landmarks  [24] or surface detection [25, 31] could enable the capture of asymmetric vertebra shapes more effectively.

Any repeatable procedural coordinate that stays fixed to the vertebral bodies can be used to establish the motion parameterization presented in this work, assuming that the vertebrae remain undeformed during FSU motion.

### Dissimilarity Study: Virtual Motion and Required Wrench Capability

The dissimilarity metrics showed different FSU poses resulting from the same commanded motion, depending on whether the intervertebral coordinates were defined preoperatively, intraoperatively, based on *Single Parent* and *Double Parent* Definition of the JCS.

#### Single Parent

For pure translations, applying *Single Parent* definition to the JCS preoperative or intraoperative FSU pose led to the same final pose of the second vertebra. A positional offset of the JCS Origin is expected to not to interfere with its directions. Pure bending virtual motions led to a distance between the positions of the second vertebra of 0.06$-$0.28 mm at $\pm 10^{\circ }$ displacement. A positional offset of the JCS Origin is expected only to affect the location of angular displacements.

We recorded no force dissimilarities in pure translation experiments using the *Single Parent* definition. However, forces led to torque dissimilarities in pure translation experiments of $\pm 2$ mm that ranged between 0.25 Nm$-$1.11 Nm. Forces acting on the JCS translate to a torque due to the lever arm of the JCS to the mounting point. The corresponding percentage (0.67$-$7.25 $\%$) remained constant for the extent of force to displacement, as expected in a linear single-axis displacement. The *Single Parent* definition of the FSU motion parameters yields the same required wrench capability (force and torque) in pure bending motions between FSU poses, regardless of whether the preoperative or intraoperative definition of the JCS is used. This is expected, as no forces are applied that could act as stray forces to disrupt the wrench.

Vertebra coordinate misalignment is not expected in pure translation experiments and does not have to be accounted for, regardless of an initial FSU pose shift. FSU pose shifts, however, lead to variation of the torque input in single-axis translation spinal loading experiments. Pure bending experiments lead to misaligning the vertebra coordinate positions, which must be accounted for when an initial FSU pose shift occurs. Due to unintuitive translational motions, we believe this poses a risk of damaging FSU tissue (e.g., supporting ligament or spinal cord). FSU pose shifts, however, do not change the wrench requirement in single-axis bending spinal loading experiments.

#### Double Parent

Applying *Double Parent* definition to the JCS preoperative or intraoperative FSU pose leads to a distance of 0.07$-$0.27 mm in pure translational motions of ± 2 mm displacements. The orientations of the second vertebra did not depend on the *Double Parent* definition of the JCS. Pure bending virtual motions resulted in a distance between the positions of the second vertebra ranging from 0.16$-$0.38 mm at a $10^{\circ }$ bending angle. We measured geodesic distances ranging from $11.48^{\circ }$ to $24.19^{\circ }$ at a $10^{\circ }$ bending angle for the FSU T9-T10 and T10-T11 segments.

We recorded force dissimilarities and torque dissimilarities in ±2 mm- pure translation displacements of 0.26$-$0.94 N (3.41$-$13.43 $\%$) and 0.01$-$0.05 Nm (2.44$-$34.97 $\%$) at ±7.5 N axial force, respectively. Using the *Double Parent* definition of the FSU motion parameters yields the same force, regardless of whether the preoperative or intraoperative FSU pose is considered. Torque dissimilarities in bending at ±7.5 Nm and $+10^{\circ }$ bending angle ranged from 0.26$-$0.95 Nm (3.41$\%$ to 13.47$\%$).

In *Double Parent* JCS Definition, vertebra position offset and angular misalignment are expected in pure translation experiments. We believe this poses a similar risk of damaging FSU tissue due to unintuitive translational motions and incorrect bending angles as in motions produced using a *Single Parent* definition. An FSU pose shift leads to variation of the force and torque input in single-axis translation spinal loading experiments. Pure bending experiments lead to changes in vertebra coordinate position and angular misalignment, which must be accounted for when an initial FSU pose shift occurs. FSU pose shifts lead to changes in torque input during single-axis bending spinal loading experiments. This would lead to wrong input torques in a pure bending experiment if not accounted for.

We are convinced that the intervertebral reference coordinate system must be defined based on the intraoperative state, which represents the equilibrium position of the FSU from which measurements will be taken. We showed that a displacement deviated depending on the status of the FSU (**Hypothesis 1**). However, accurately capturing the intraoperative state may be challenging for in vivo spinal loading experiments, depending on the available intraoperative imaging modalities. We showed that differences in the JCS definition affect both the location of the deflected vertebra (**Hypothesis 2**). The wrench required to achieve a given displacement in FSU loading experiments using pure translation and bending criteria, especially when a vertebral shift occurs before the experiment, was demonstrated (**Hypothesis 3**). Our findings indicate that an FSU motion derived from a *Double Parent* definition of the JCS is more susceptible to pose changes than motion based on a *Single Parent* JCS definition.

### Developed Convention

We showed that minute changes to the convention may implicate load-to-displacement behavior to a relevant magnitude, thus highlighting the importance of an unambiguous systematic motion parametrization. Assessing biomechanical properties through wrench/displacement measurements in scoliotic FSUs depends on the measurement regime. In an impedance measurement regime, the intervertebral reference coordinate system position and JCS must be defined before the measurement. We believe that for an impedance measurement, the *Single Parent* definition was used due to its simplicity in SLS studies. However, we believe that for a system to operate in impedance and admittance measurement regimes, the *Single Parent* definition is insufficient. We would not assume that anatomically meaningful joint axes between two vertebrae, around which a force/torque profile should be applied, would remain constant relative to only one of them. In such an admittance measurement regime, where a force/torque profile is enforced, potentially unsafe or unintuitive movements are measured, the JCS should be continuously tracked and possibly recalculated at each time step. Here, a discrete shift in the intervertebral reference position and frame may be considered a small, yet notable, change in the FSU pose that would result in a mismatch of position and wrench requirement. Furthermore, we believe that with a *Double Parent* Definition of the JCS, any effect of the cranial vertebra that may affect the load/displacement or displacement/load properties in pathological FSUs could be investigated. For better generalization, e.g., a convention that works for impedance and admittance regimes and strongly asymmetrical or skewed vertebrae, we recommend using the *Double Parent* definition for the JCS. Our findings indicate that a produced FSU motion and required input wrench based on a *Double Parent* definition are susceptible to FSU pose changes. Therefore, the equilibrium state must be known before an in vivo spinal loading experiment for an accurate and safe readout.

Our contribution to the field of spine biomechanics is the development of a systematic convention for intervertebral motion parameterization of FSU directions, which does not yet exist in the field. We approach this with a combination of vertebral coordinate generation based on vertebral body-centered anatomical landmarks and with rigid body and virtual robotic joint modeling.

This convention was applied to analyze the changes in FSU pose from preoperative to intraoperative stages in AIS cases by comparing preoperative to intraoperative imaging data. Our findings demonstrated that the initial state of the FSU relevantly influenced the resulting motion and determined the required wrench in a loading scenario. Additionally, we demonstrated that even a subtle variation in FSU joint definition—such as the difference between the *Single Parent* and *Double Parent* definitions of the JCS—could meaningfully impact the generated motion and the required wrench capability of an actuation device.

Our novel kinematic framework for the FSU can be practically applied to any in vivo robotic system designed for spinal loading. Consistently following our convention would enable safer, more intuitive, and more reproducible assessments under both impedance- and admittance control modes for single-axis displacements relative to the FSU’s equilibrium state. We recommend using our convention with the *Double Parent* definition for generalizable and robust FSU assessments. Particularly for scoliotic segments, such a convention is required to ensure reliable biomechanical evaluation and support future surgical decision-making in AIS.

## Data Availability

All data, material, and code used for this study are publicly available under the title *“FAErb_2025_Importance of a Systematic Intervertebral Motion Parametrization for in vivo Assessment of Spine Biomechanics”* via DOI: 10.21227/0mws-5503.
